# A Global Regional Comparison of the Risk of Breast Cancer in Woman Using Oral Contraceptives—Systematic Review and Meta-Analysis

**DOI:** 10.3390/cancers16234044

**Published:** 2024-12-02

**Authors:** Agnieszka Drab, Krystian Wdowiak, Wiesław Kanadys, Maria Malm, Joanna Dolar-Szczasny, Grzegorz Zieliński, Mariola Borowska, Urszula Religioni

**Affiliations:** 1 Department of Medical Informatics and Statistics, Medical University of Lublin, 20-059 Lublin, Poland; krystianrwdowiak@interia.eu (K.W.); maria.malm@umlub.pl (M.M.); 2 Specialistic Medical Center Czechow, Gynecology Unit, 20-848 Lublin, Poland; wieslaw.kanadys@wp.pl; 3 Department of General and Pediatric Ophthalmology, Medical University of Lublin, 20-093 Lublin, Poland; joannaszczasny@op.pl; 4 Department of Sports Medicine, Medical University of Lublin, 20-093 Lublin, Poland; grzegorz.zielinski@umlub.pl; 5 Cancer Epidemiology and Primary Prevention Department, Maria Sklodowska-Curie National Research Institute of Oncology, 02-781 Warsaw, Poland; mariola.borowska@nio.gov.pl; 6 School of Public Health, Centre of Postgraduate Medical Education of Warsaw, 00-041 Warsaw, Poland; urszula.religioni@gmail.com

**Keywords:** breast cancer, oral contraceptives, geographic region, meta-analysis

## Abstract

**Background**: Incidence of breast cancer (BrCa) may be correlated with country development, with a rise in cases anticipated in regions of the world that are currently undergoing an economic transformation. Herein, differences with regard to the occurrence of breast cancer between individual countries may depend on the distribution of risk factors, the level of early detection, also ethnicity and race, as well as clinical characteristics. The aim of our study was to identify and then investigate observational studies in which the risk of breast cancer was associated with the use of oral hormonal contraceptives (OCs), with particular emphasis on geographic region, and to conduct a systematic review and meta-analysis of the obtained data. **Methods**: RR (relative risk) was calculated and displayed in forest plots for visual interpretation. Accordingly, 74 studies involving a total of 198,579 women were eligible for inclusion in the meta-analysis. This is the first meta-analysis to comprehensively summarize the evidence between OC use and BrCa risk in connection with geographical region. **Results**: The cumulative results of the meta-analysis for specific parts of the world are: Africa (RR = 1.16, *p* = 0.216) and the Americas (RR = 1.03, *p* = 0.597); Asia (RR = 1.29, *p* = 0.014); European countries (RR = 1.01, *p* = 0.904); and Middle East countries (RR = 1.29, *p* = 0.043). Subgroup analyses showed an increased risk of BrCa for the analyzed variables that depended upon the geographical region. **Conclusions**: Our meta-analysis suggests that OC use may be associated with a higher BrCa risk, although a statistically significant association was not found for all geographical regions of the world.

## Simple Summary

Oral Contraceptives (OCs) may play a role in the development of breast cancer. A meta-analysis was undertaken using a DerSimonian–Laird random effects model to investigate the association between OCs use and breast cancer (BrCa) risk by geographic region of the world. A systematic review was performed (MEDLINE, EMBASE, and the Cochrane Library) from1995 to 2023. Our meta-analysis suggests that OC use may be associated with a higher BrCa risk, although a statistically significant association was not found for all global geographical regions.

## 1. Introduction

Breast cancer (BrCa) is one of the most common malignancies among women throughout the world and is the major cause of most cancer-related deaths. In the general population, the cumulative lifetime risk for BrCa is 8–10% [1,2]. Overall, in 2020, 2,26 mln new incidences were diagnosed for this malignant tumor. Out of 1000 women by the age of fifty years, as many as two will receive a BrCa diagnosis [3,4]. The incidence of this cancer is correlated with development, with a rise in cases anticipated in regions of the world that are currently undergoing an economic transformation [5,6,7]. The literature indicates that there are many factors that increase the risk of BrCa, including nulliparity or late age at first birth, early menarche, late menopause, infertility, and the use of exogenous hormones. Environmental factors, such as obesity, selected dietary habits, alcohol consumption, smoking, and a lack of physical activity, may also affect the increased risk of BrCa [8,9,10,11,12].

The majority of BrCa cases are sporadic and most of the familial risks of breast cancer remain unexplained. However, it is estimated that approximately 5 to10% have a genetic predisposition related to, inter alia, family cancer histories for first-degree relatives or genetic mutation carrier status [13,14]. BrCa and ovarian cancer are both in the top 10 of the most common and deadly tumors for women and, among the risk factors for the development of these cancers, genetic predisposition plays an important role [15].

BrCa has a hereditary basis. BRCAs (BReast CAncer genes) 1 and 2 are tumor suppressor genes with pivotal roles in the development of breast and ovarian cancers [16,17,18]. Several additional genes have recently been associated with the above-mentioned genes. Inherited mutations in these genes predispose to breast cancer with varying penetration estimates. These genes can be classified as highly penetrant genes with a lifetime risk >30% (TP53, PTEN, CDH1, STK11, and PALB2) and moderately penetrant genes with a 17–30% risk (CHEK2, ATM, BARD1, BRIP1, NBN, NF1, RAD51D, and MSH6) [19,20,21]. BrCa subtypes are commonly grouped into four categories based on the immunohistochemical expression of hormone receptors: human epidermal growth factor receptor positive (HER2+), progesterone receptor positive (PR+), estrogen receptor positive (ER+), and triple negative (TNBC) [22]. Furthermore, differences in the risk of developing breast cancer have been observed in relation to individual BrCa subtypes [22]. Subtypes have a different biology with accumulating evidence of different risk factors [23]. Molecular BrCa classification is useful not only for prognosis, but also for targeted therapy. Knowing the subtype of breast cancer can help clinical practice to establish the best treatment [24,25,26,27].

Studies of BrCa risk among women who use oral contraceptives (OCs) in general showed conflicting results: from no increase in risk, to a 20%–30% elevation in risk. Our previous meta-analyses showed that the taking of OCs in the general female population was associated with a moderately, statistically significant increased risk of BrCa, compared with those who do not used this contraceptive (OR = 1.15, 95% CI: 1.01 to 1.31, *p* = 0.0358), [28,29].

The aim of our study was to identify published observational studies in which the risk of breast cancer was associated with the use of oral hormonal contraceptives with particular emphasis on global geographic regions, and, subsequently, to conduct a systematic review and meta-analysis of the obtained data.

## 2. Materials and Methods

This study is reported as per the Preferred Reporting Items for Systematic Reviews and Meta-Analyses (PRISMA) guideline [30]. This systematic review and meta-analysis study was registered in PROSPERO—International prospective register of systematic reviews (with registry ID: CRD42023393989).

### 2.1. Search Strategy

We searched for articles written in English using the medical research databases of PubMed (MEDLINE), Embase, and the Cochrane Library to identify studies published from January 1995 up to February 2023 related to our work. Studies were searched for using the keywords: [‘breast cancer’ or ‘breast neoplasm’ or ‘breast carcinoma’] AND [‘oral contraceptives’ or ‘oral contraceptive pills’ or ‘birth control pills’] AND [‘cohort’ or ‘case-control’]. To ensure full access to relevant studies, we also analyzed the references of previously published review articles, meta-analyzes, and other publications. In addition, we searched the potentially eligible bibliographies of relevant articles for the purpose of completeness.

### 2.2. Study Selection

Inclusion criteria. The studies that met the following criteria were included in the meta-analysis: articles assessing the relationship between OC use and BrCa risk; case–control and cohort study design; and studies using raw data. In cases in which a study was published in different phases or where the same data from a trial were duplicated in more than one study, the most complete study was included in the analysis.

Exclusive criteria. Studies were excluded sequentially based on the following criteria: inappropriate designs (cross-sectional study, women with BrCa being the control group); reviews, commentaries, editorials, or guidelines; data for analysis were not available; and articles published in languages other than English.

### 2.3. Data Extraction

The data extraction sheet consisted of the author’s name, publication’s year, study type, the country/region in which the study was performed, follow-up duration, the number of patients, and source of cases, information on the usage of OCs in both groups, and BrCa incidence depending on menarche, parity, breastfeeding, family history of BrCa, body mass index (BMI), and tobacco smoking.

### 2.4. Assessment of Study Quality

The Newcastle–Ottawa scale (NOS) was used to evaluate the quality of case–control studies and cohort studies included to the meta-analysis [31]. A system of stars/points was given to the eligible categories. The NOS for cohort studies mainly considers three methods: selection, comparability, and outcome. Each numbered item in the selection and outcome categories can earn up to 1 star, while comparability can receive up to 2 stars. For case–control studies, the NOS includes the following categories: selection, comparability, and exposure. Each numbered item in the selection and exposure categories can receive up to 1 star, while comparability can receive a maximum of 2 stars. A maximum of 9 stars/ score) can be given to determine the risk of bias [31]. Two researchers (ADrab and W.Kanadys) independently rated the methodological quality of the included studies. In the case of disagreements, a decision was reached by consulting a third investigator (MMalm).

### 2.5. Statistical Analysis

BrCa risk after OCs intake was compared to of BrCa risk without exposure to OCs during the same time period in individual regions of the world. The strength of the association between OC use and BrCa risk was calculated with the risk ratio (RR) and respective 95% confidence interval (CI) [32]. Forest plots were used to visually assess the RR estimates and CIs across studies. A DerSimonian–Laird random-effects model was used to calculate the pooled RR and 95% CI [33]. Heterogeneity across studies was evaluated by the I^2^ index and the Q test. I^2^ values of ≤30%, 31–74%, and ≥75% were regarded as, respectively, representing low, moderate, and high heterogeneity between studies [34]. Publication bias was assessed by the Begg’s rank correlation test and Egger’s regression test [35,36]. Additional analyses were conducted for individual geographical areas to identify the potential impact of modifying factors: age at menarche, parity, breastfeeding, body mass, index, cigarette smoking, and family history of breast cancer on increase in breast cancer risk. For all tests, a *p* value of less than 0.05 was considered statistically significant. Statistical analysis was performed using STATISTICA 13.3 software (StatSoft, Krakow, Poland) and Microsoft Excel.

## 3. Results

**Figure 1:**
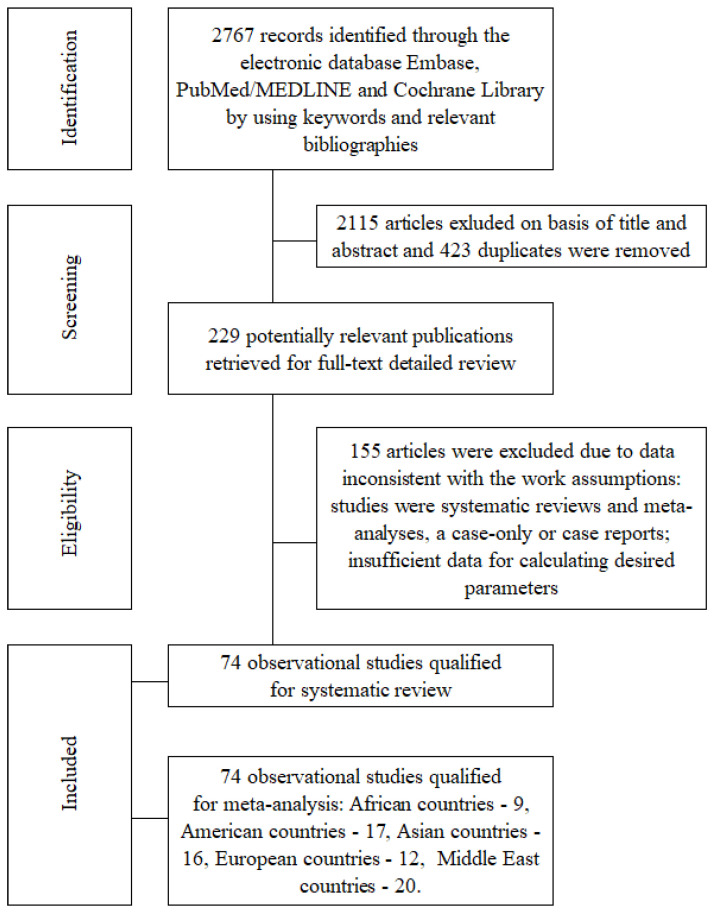
Flow diagram of studies exploring the association between OC and BrCa risk in accordance with the PRISMA guidelines.

According to the search strategy, a total of 2767 citations were identified, 2538 of which were rejected after the initial reviewing of the titles, abstracts, and duplicates, leaving 229 articles for a full-text review. In turn, 155 studies were excluded based on the design and specific exposure. We identified three studies for the Australia and Oceania regions, but this is not a representative sample for meta-analysis. Of the remaining, 74 studies were included in the meta-analysis. All were case–control or cohort studies that evaluated the relationship between OC intake and BrCa risk in individual regions of the world (Figure 1).

The meta-analysis included 74 studies reported in 74 articles (Table 1). Here, 198,579 women, 64,483 in the group with BrCa and 134,096 in the control/population group (control for case–control studies and population for cohort studies), were included in the meta-analysis. All studies were included in accordance with the NOS scale. The NOS value was in the range of 4–8 points, and the average score was 5.82 points for included studies. Twenty-eight studies (37.84%) were regarded as high-quality studies (NOS ≥ 7) and forty-six studies (62.16%) as moderate-quality studies (NOS ≥ 4). The source of subjects, according to the information provided in the manuscripts, was: hospital, community, clinic, or population.

### 3.1. Effects of Oral Contraceptive Use on Breast Cancer Risk in African Countries

**Figure 2:**
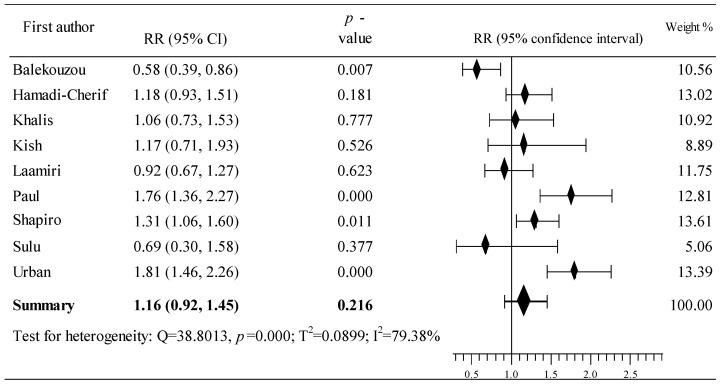
Forest plots showing the summary RR and 95% CI studies conducted in African countries [37,38,39,40,41,42,43,44,45].

The analysis of the impact of the taking of OCs on the risk of developing BrCa in women from the African region was based on nine articles [37,38,39,40,41,42,43,44,45]. The research included data from 9322 participants (case group: 3530; population group: 5792, respectively); including 1663 and 2483 women who use OCs. The analysis of BrCa risk showed an increase in six studies [38,39,40,43,44,45], where this increase was statistically significant in three studies [38,43,44]. Three studies demonstrated a reduction in the risk of BrCa [37,41,42], including one study with a statistically significant decrease [41]. The cumulative result of the meta-analysis is RR = 1.16, 95% CI: 0.92 to 1.45, *p* = 0.216, with relatively high heterogeneity (I^2^ = 79.38%) (Figure 2). Begg’s and Egger’s tests showed no evidence of publication bias: tau b = –0.333, z = –1.051, *p* = 0.293 and b0 = –3.694, 95% CI: –8.080 to 0.691, t = –1.992, *p* = 0.087.

### 3.2. Effects of Oral Contraceptive Use on Breast Cancer in Countries in the Americas

**Figure 3:**
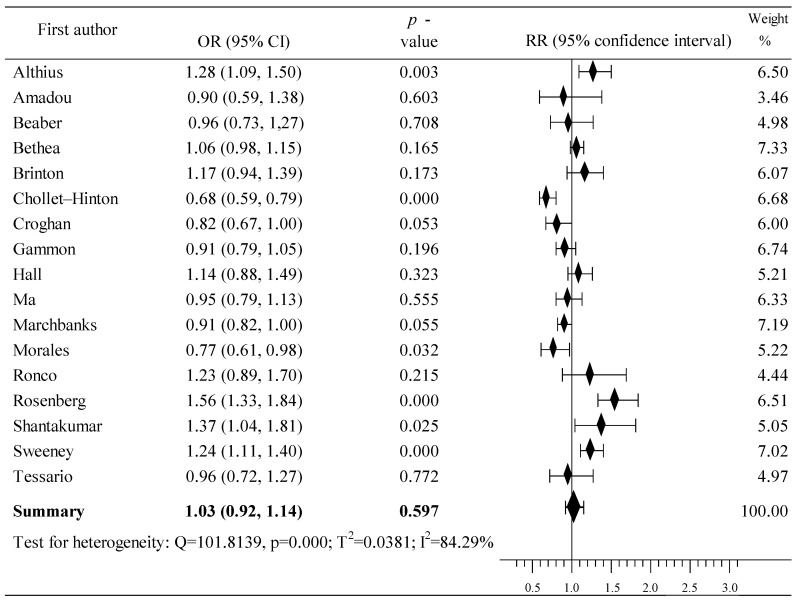
Forest plots showing the summary RR and 95% CI studies conducted in American countries [46,47,48,49,50,51,52,53,54,55,56,57,58,59,60,61,62].

Seventeen studies from certain countries located in the Americas assessed the relationship between the use of OCs and the risk of BrCa [46,47,48,49,50,51,52,53,54,55,56,57,58,59,60,61,62]. The analysis covered 58,648 women, including 21,849 in the case group and 36 799 in the population group; 15,943 and 24,522 were taking birth control pills in both groups, respectively. Increased risk was reported in eight studies [46,48,52,55,56,57,58,59], including statistically significant ones in four studies [55,56,57,58,59]. In turn, a risk reduction was shown in nine studies [47,49,50,51,53,54,60,61,62], including two that were statistically significant [47,51]. The overall result of the meta-analysis is associated with a slight increase in the risk of BrCa after taking OCs, RR = 1.03, 95% CI: 0.92 to 1.14, *p* = 0.597, with high heterogeneity, I^2^ = 84.29% (Figure 3). Begg’s test (tau b = –0.124, z = –0.643, *p* = 0.520) and Egger’s test (b0 = 0.077, 95% CI: –3.230 to 3.385, t = 0.050, *p* = 0.961) did not show evidence of publication bias.

### 3.3. Effects of Oral Contraceptive Use on Breast Cancer in Countries in Asia

**Figure 4:**
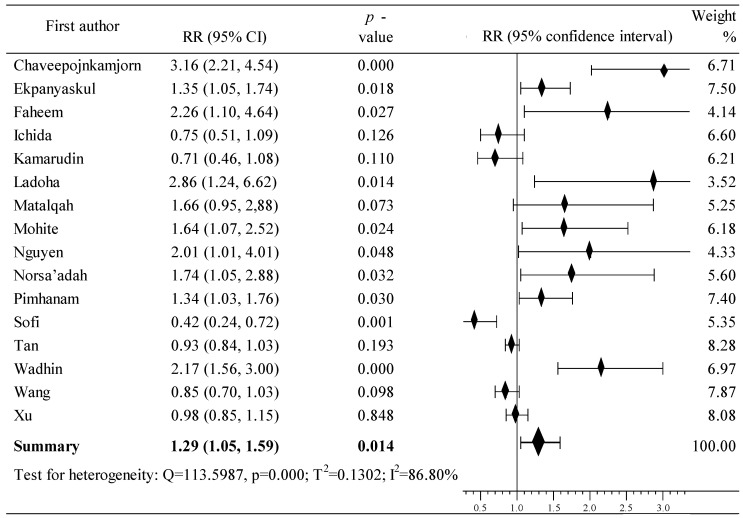
Forest plots showing the summary RR and 95% CI studies conducted in Asian countries [63,64,65,66,67,68,69,70,71,72,73,74,75,76,77,78].

The risk of BrCa in Asia was investigated based on the data from sixteen studies conducted in several Asian countries [63,64,65,66,67,68,69,70,71,72,73,74,75,76,77,78]. A total of 31,963 women participated in the study: 9655 in the case group and 22,308 in the population group. In both group, OCs were used by 2732 and 6224 participants. Increased risk of BrCa was observed in ten studies [64,66,67,70,71,72,73,75,76,78], including nine where the increase in risk was statistically significant [64,66,67,70,71,72,75,76,78]. Six studies showed a lowering of cancer risk after OC use [63,65,68,69,74,77], including one study in which the reduction was significant [65]. Meta-analysis for Asian countries showed that the use of oral contraceptives resulted in a statistically significant increase in the risk of BrCa development: RR = 1.29, 95% CI: 1.09 to 1.59, *p* = 0.014, I^2^ = 86.80% (Figure 4). Begg’s (tau b = 0.205, z = 0.976, *p* = 0.329) as well as Egger’s (b0 = 2.316, 95% CI: –0.683 to 4700, t = 0.281, *p* = 0.056) tests showed no publication bias.

### 3.4. Effects of Oral Contraceptive Use on Breast Cancer in Europe

**Figure 5:**
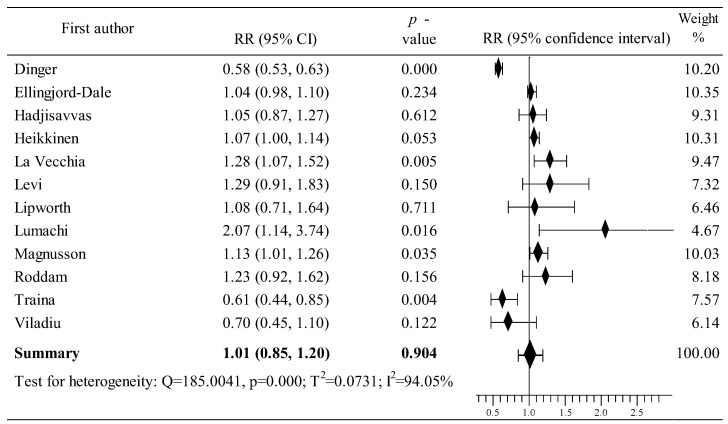
Forest plots showing the summary RR and 95% CI studies conducted in European countries [79,80,81,82,83,84,85,86,87,88,89,90].

As shown in Figure 5, changes in the risk of BrCa in users of OCs was assessed in twelve studies conducted in particular countries within Europe. The study was based on data from 84,967 participants, including 22,781 women in the case group and 62,186 in the population group [79,80,81,82,83,84,85,86,87,88,89,90]. In both groups, OCs were used by 11,920 and 36,383 women. Nine studies reported an increase in the risk of BrCa [79,80,81,82,83,85,86,89,90], including three in which the increase was statistically significant [82,85,89]. In turn, three studies showed a decrease in the risk of BrCa after the use of OCs [84,87,88], including two studies where the reduction was statistically significant [84,87]. Ultimately, the results of the meta-analysis are associated with a slight increase in the risk of BrCa: RR = 1.01, 95% CI: 0.85 to 1.20, *p* = 0.904; with high heterogeneity I^2^ = 94.05% (Figure 5). Begg’s and Egger’s tests showed no evidence of publication bias: tau b = −0.214, z = −0742, *p* = 0.458 and b0 = 0.577, 95% CI; −3.998 to 5.153, t = 0.281, *p* = 0.784.

### 3.5. Effects of Oral Contraceptive Use on Breast Cancer in Countries in the Middle East

**Figure 6:**
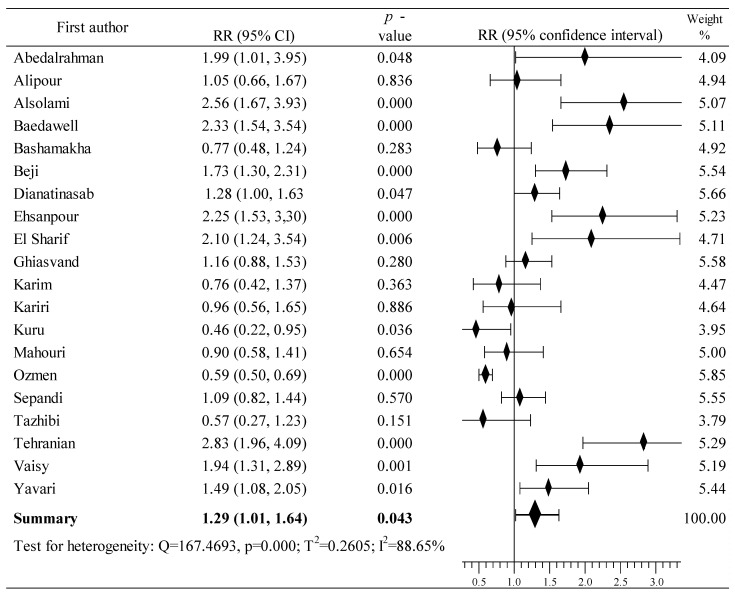
Forest plots showing the summary RR and 95% CI studies conducted in Middle East countries [91,92,93,94,95,96,97,98,99,100,101,102,103,104,105,106,107,108,109,110].

The effect of oral contraceptive use on the risk of developing breast cancer was determined on the basis of data from twenty studies conducted in several Middle East countries [91,92,93,94,95,96,97,98,99,100,101,102,103,104,105,106,107,108,109,110]. The study involved 25,239 women, including 5820 in the case group and 19,419 in the population group; contraception was used by 2732 and 6224 participants, respectively. An increase in the risk of BrCa was observed in thirteen studies [91,92,93,94,96,97,100,101,103,104,105,108,109], of which 10 displayed a statistically significant increase [91,93,94,96,97,100,103,105,108,109]. BrCa risk decrease was reported in seven studies [95,98,99,102,106,107,110], and in one it was statistically significant [106]. The result of the meta-analysis shows a statistically significant increased risk of BrCa: RR = 1.29, 95% CI: 1.08 to 1.64, *p* = 0.043, I^2^ = 88.65% (Figure 6). Begg’s test was significant for publication bias (tau b = –0.346, z = –2.007, *p* = 0.045), but not Egger’s test: = b0 = 2.735, 95% CI: –0.672 to 6.141, t = 1.686, *p* = 0.109.

### 3.6. The Impact of Modifying Factors on Breast Cancer Risk

Separate analyses were conducted for each individual geographical area so as to identify the impact of modifying factors on breast cancer risk (Table 2). Herein, the occurrence of menarche at <12 years compared with ≥12 years was associated with a non-significant increase in the risk of BC among women from the Middle East (*p* = 0.056), but also from Asia (*p* = 0.110) and the Americas (*p* = 0.753). A non-significant reduction in risk was seen among European (*p* = 0.970) and African (*p* = 0.452) women. The analysis of parity (nulliparous vs. parous) showed a statistically significant increase in breast cancer risk in Africa (RR = 2.206, *p* = 0.003) and Europe (RR = 1.337, *p* = 0.000), and was non-significant among women from the Middle East (*p* = 0.104), the Americas (*p* = 0.499), and Asia (*p* = 0.669). A statistically significant increase in the risk of breast cancer related to breastfeeding (no/yes) was observed in women from Africa (RR = 2.112, *p* = 0.030), the Middle East (RR = 1.878, *p* = 0.007), the Americas (RR = 1.123, *p* = 0.047), and Asia (RR = 1.864, *p* = 0.000), as well as being non-significant among women from Europe (*p* = 0.208). It has been observed that the risk of developing breast cancer may increase with an increasing body mass index (BMI) from a normal weight to overweight and obesity. An increase in BMI from ≤25 to 26–29 kg/m^2^ was associated with a statistically significant increase in breast cancer incidence in Africa (RR = 1.420, *p* = 0.008), and a non-significant increased risk of cancer among women from Asia (*p* = 0.288), the Americas (*p* = 0.505), and the Middle East (*p* = 0.770). There was also a non-significant slight reduction in the risk of BrCa in European women (*p* = 0.570). Also evident was a significant increase in BrCa risk with an increase in BMI from ≤25 to ≥30 kg/m^2^ in African women (RR = 2.249, *p* = 0.033), and a clear but not statistically significant increase in cancer incidence in women from Asia (*p* = 0.103), Europe (*p* = 0.8150), and the Middle East (*p* = 0.233). The relationship between cigarette smoking and the risk of breast cancer in women in different geographic regions has similar trends: Africa (*p* = 0.193; this is based on only two works), the Americas (*p* = 0.592), Asia (*p* = 0.089), Europe (*p* = 0.524), and the Middle East (*p* = 0.297). Family history of breast cancer among women in different regions of the world has a statistically significant impact on the increase in the incidence of this cancer: Africa (RR = 3.897, *p* = 0.000), the Americas (RR = 1.780, *p* = 0.000), Asia (RR = 1850, *p* = 0.001), Europe (RR = 2.014, *p* = 0.001), and the Middle East (RR = 1.804, *p* = 0.004).

## 4. Discussion

This is the first meta-analysis to comprehensively summarize the evidence of OC use and BrCa risk in connection with geographical region. Cumulative results of meta-analysis for specific parts of the world are: Africa (RR = 1.16, *p* = 0.216), and the Americas (RR = 1.03, *p* = 0.597)—both locations show an insignificant increase in BrCa risk. An analysis of Asian statistics showed that use of OCs resulted in a statistically significant increase in BrCa risk: RR = 1.29, *p* = 0.014. In the assessed European countries, this was associated with a statistically insignificant increase in the risk of BrCa: RR = 1.01, *p* = 0.904, while Middle East countries revealed a statistically significant increase risk of BrCa: RR = 1.29, *p* = 0.043. Subgroup analyses showed an increased risk of breast cancer (BrCa) for analyzed variables depending on geographical region. Our results indicate that, globally, family history of BrCa for women has a significant impact on the increase in the incidence of this cancer. Here, the statistics are: Africa (RR = 3.897, *p* = 0.000), the Americas (RR = 1.780, *p* = 0.000), Asia (RR = 1.850, *p* = 0.001), Europe (RR = 2.014, *p* = 0.001), and the Middle East (RR = 1.804, *p* = 0.004). The analysis of parity (nulliparous vs. parous) showed an increase in BrCa risk in Africa (RR = 2.206, *p* = 0.003) and Europe (RR = 1.337, *p* = 0.000). Statistically significant increase in the risk of BrCa was related to breastfeeding (no/yes) as observed in women from Africa (*p* = 0.030), the Middle East (RR = 1.878, *p* = 0.007), the Americas (RR = 1.123, *p* = 0.047), and Asia (RR =1.864, *p* = 0.000). An increase in BMI from ≤25 to 26–29 kg/m^2^ was associated with a statistically significant increase in breast cancer incidence in women from Africa (RR = 1.420, *p* = 0.008). Similar results were shown in our previous meta-analysis [28] conducted for studies in 1960–2010, where the study demonstrated no significant increase in BrCa risk among women (1.01, 0.95–1.01, *p* < 0.688). Our second meta-analysis covering the period 2009–2020 with 42 studies and a total of 110,580 enrolled women showed that the use of OCs was associated with a significantly increased risk of BrCa in general, OR = 1.15, 95% CI: 1.01 to 1.31, *p* = 0.0358 [29].

The current meta-analysis examines the effects of contraceptives on BrCa by geographical region. There is still a lack of studies that differentiate cancer incidence in specific countries in the context of contraceptive use. Meta-analysis provided by Soroush et al. about the relationship between OC use and BrCa among Iranian women indicates that using birth control pills increases the risk of BrCa in Iran up to 1.52 times. In such data, the estimate of OR for the effect of OC use was 1.521 (CI = 1.25–1.85), and demonstrates that the intervention group had a higher chance of survival (52%) compared to the control group (*p* = 0.001), [111]. Furthermore, meta-analysis of studies involving 28,776 Southeast Asian women found a slight increase in BrCa risk with oral contraceptives’ application ≤5 years with an OR = 1.21 (95% CI 0.96–1.52, *p* > 0.05), while a higher risk of breast cancer was found in women with an oral contraceptive application >5 years with an OR = 2.66 (95% CI 1.79–3.94, *p* < 0.00001), [112].

Breast cancer (BrCa) incidence rates have historically been several-times higher in the United States than in China or Japan [113], and the scientific literature from before 2000 indicates that there are significant disparities in contraceptive use and breast cancer incidence for different regions of the world [114]. This is due to migration, economic development, and levels of health awareness. However, the current research shows that there has been an increasing trend in the use of contraceptives from less than approximately 10% to more than 50% of the population in several developing countries, and the risk rates are constantly growing in Asia and in the Middle East [115].

The observed differences in the occurrence of BrCa between individual countries may depend on the differences in the incidence of this cancer and the distribution of risk factors, as well as in the rate of early detection of this cancer in these countries. They may also differ significantly depending on ethnicity and race, clinical characteristics, and prognosis [5,6,7]. The study of Xie et al. showed breast cancer incidence that is related to OC application in several ethnicities [116]. Herein, oral contraceptive use (HR = 1.09, 95%, CI = 1.01, 1.18) is associated with an increased risk of breast cancer in non-Hispanic Caucasians alone. However, the long-term use of menopausal hormone therapy (more than five years) was associated with an increased risk of breast cancer in both the non-Hispanic Caucasian (HR = 1.44, 95% CI = 1.31, 1.59) group and the non-Hispanic Asian/Pacific Islander (HR = 1.98, 95% CI = 1.23, 3.20) group, but not in other race/ethnic groups [116].

Study limitations, such as risk measures and design heterogeneity, may have contributed to the reported results. Studies, including summary estimates, are vulnerable to various types of bias. Due to a lack of data, this study did not account for important potential confounders—physical activity, diet/nutrition, or defined times that pill self-administration was begun and ended. In addition, the use of different OCs and other genetic factors were not explored. Another potential limitation of the study is the lack of a uniform definition of ‘ever’ use of OCs. The timing of OC use and its effect on breast cancer risk could also have been a source of bias. Unfortunately, most studies did not include this information. Additional investigations of this association are warranted, especially to assess the effect of modern OCs and to determine whether the elevated risk among women concerns long-term use. We are aware that drawing final conclusions from the results of our meta-analysis requires caution, given the number of limitations encountered in its construction. Of note, we limited the search to studies published in English, and these were identified through electronic databases. The possibility of not viewing all the publications on this topic may have had an effect on the value of the results. Another limitation may be the source of subjects extracted from the included studies. Different regions classify the majority of studies as clinic, while in others there is a higher prevalence of population studies. This could have influenced the results of individual geographic regions and the heterogeneity of the studies. Another limitation is the lack of information on women’s place of residence in the included studies. An additional limitation for Turkey was the difficulty of classifying its geographic region. The included studies did not contain information about which part of the country the women came from [106,108,110]. Due to the fact that Turkey is considered as a Middle East country and the European part covers less than 3% of the country, it was included in the meta-analysis as a Middle East country.

## 5. Conclusions

Our meta-analysis suggests that OC use may be associated with a higher BrCa risk, although a statistically significant association was not found for all global geographical regions. The study showed how important it is to have access to a family history of breast cancer for all women in the world.

## Figures and Tables

**Table 1:** Summary of characteristics of the included studies.

First Author Pub Year [References]	Country	Recruitment Years	Number of Cases (% OC Use)	Number of Controls Subjects (% OC Use)	Age Range Years	Study Design	Source of Subjects	NOS Score
**A. African countries**								
Sulu 2022 [37]	Democratic Republic of the Congo	2014–2019	160 (93.7)	320 (95.6)	26–75	cohort	Hospital	7
Paul 2020 [38]	Cameroon	2012–2018	297 (50.5)	1158 (36.7)	48.5 ± 2.6	case-control	Hospital	8
Hamadi-Cherif 2020 [39]	Algeria	2012–2017	547 (63.1)	543 (59.1)	28–77	case-control	Hospital	4
Khalis 2018 [40]	Morocco	2014–2015	237 (62.2)	237 (61.2)	45–54	case-control	Hospital	6
Balekouzou 2017 [41]	Central African Republic	2003–2015	174 (28.9)	348 (41.1)	45.8 ± 13.6	case-control	Population	6
Laamiri 2015 [42]	Morocco	2008–2010	400 (74.5)	400 (76.0)	45.8 ± 11.1	cohort	Hospital	5
Urban 2015 [43]	South Africa	1995–2006	1112 (23.0)	1102 (14.2)	18–79	case-control	Hospital	4
Shapiro 2000 [44]	South Africa	1994–1997	484 (45.5)	1625 (39.0)	20–54	case-control	Hospital	7
Kishk 1999 [45]	Egypt	no data	129 (57.4)	129 (61.2)	44.5	cohort	Hospital	5
**B. Countries from the Americas**								
Brinton 2018 [46]	USA	1990–1992	1031 (72.5)	919 (69.7)	<54	case-control	Population	6
Chollet-Hinton 2017 [47]	USA	2005	1589 (80,7)	5137 (86.1)	22–75	cohort	Population	7
Bethea 2015 [48]	USA	1993–2011	2891 (55.3)	10,044 (53.9)	20–75	case-control	Population	7
Beaber 2014 [49]	USA	2004–2009	985 (87.9)	882 (83.3)	20–44	cohort	Population	7
Amadou 2013 [50]	Mexico	2004–2007	263 (17.9)	314 (19.4)	35–64	case-control	Hospital	6
Morales 2013 [51]	Puerto Rico	2005–2009	462 (48.5)	649 (55.0)	56.4 ± 12.6	case-control	Hospital	6
Ronco 2012 [52]	Uruguay	2004–2010	251 (69.7)	497 (65.2)	<30–50≥	case-control	Hospital	8
Ma 2010 [53]	USA	1994–1998	1197 (78.8)	2015 (79.6)	36–64	case-control	Community	8
Croghan 2009 [54]	USA	1993–2003	531 (66.1)	2150 (70.4)	53.7 ± 15.02	case-control	Clinic	6
Rosenberg 2009 [55]	USA	1993–2007	907 (52.6)	1711 (41.5)	25–69	case-control	Hospital	7
Shantakumar 2007 [56]	USA	1996–1997	468 (72.9)	500 (66.2)	20–50	cohort	Population	6
Sweeney 2007 [57]	USA	1999–2004	2303 (64.9)	2513 (59.8)	<64	cohort	Population	7
Hall 2005 [58]	USA	1993 –2001	957 (85.5)	763 (83.7)	20–49	cohort	Population	5
Althuis 2003 [59]	USA	1990–1992	1640 (77.4)	1492 (72.8)	20–44	case-control	Population	5
Gammon 2002 [60]	USA	1996–1997	1505 (43.6)	1556 (45.9)	<45–75+	case-control	Population	7
Marchbanks 2002 [61]	USA	1994–1998	4575 (76.1)	4682 (78.1)	35–64	cohort	Population	5
Tessaro 2001 [62]	Brasil	1995–1998	340 (74.4)	1020 (75.2)	20–60	case-control	Hospital	6
**C. Asian countries**								
Tan 2018 [63]	Malaysia	2002–2016	3387 (27.6)	58.0	40–74	case-control	Population	4
Wahidin 2018 [64]	Indonesia	2018	381 (35.4)	381 (20.2)	40–49	case-control	Hospital	4
Sofi 2018 [65]	India	2015–2017	195 (12.3)	191 (25.1)	45.0 ± 10.0	case-control	Hospital	7
Chaveepojnkamjorn 2017 [66]	Thailand	2013–2014	257 (65.0)	257 (37.0)	25–44	case-control	Hospital	7
Nguyen 2016 [67]	Vietnam	2007–2013	291 (8.6)	291 (4.5)	24–65	case-control	Hospital	7
Wang 2016 [68]	Hong Kong SAR	2011–2015	923 (32.6)	918 (36.3)	56.0 ± 11.8	cohort	Hospital	6
Ichida 2015 [69]	Japan	2007–2013	155 (23.2)	12,223 (26.8)	20–69	case-control	Clinic	6
Mohite 2015 [70]	India	2009–2011	217 (31.8)	217 (22.1)	40–49	case-control	Hospital	4
Pimhanam 2014 [71]	Thailand	2007–2011	444 (45.9)	444 (38.7	45.8 ± 10.1	case-control	Hospital	4
Ladoha 2011 [72]	India	2008–2009	207 (10.1)	211 (3.8)	28–78	case-control	Hospital	5
Matalqah 2011 [73]	Malaysia	2009–2010	150 (26.7)	150 (18.0)	52.8 ± 1.1	case-control	Population	7
Xu 2011 [74]	China	1996–1998 2002–2005	2073 (20.2)	2083 (20.4)	49.5 ± 8.3	cohort	Population	7
Ekpanyaskul 2010 [75]	Thailand	2002–2004	516 (42.0)	516 (34.9)	46.9 ± 10.6	case-control	Hospital	6
Faheem 2007 [76]	Pakistan	2005	132 (18.2)	145 (9.0)	42.4	case-control	Hospital	6
Kamarudin 2006 [77]	Malaysia	2004–2004	188 (34.4)	183 (42.6)	48.7	cohort	Hospital	6
Norsa’adah 2005 [78]	Malaysia	2000–2001	147 (36.1)	147 (24.5)	26–70	case-control	Hospital	5
**D. European countries**								
Ellingjord-Dale 2017 [79]	Norway	2006–2014	5050 (51,6)	24,343 (50.7)	50–69	case-control	Population	8
Heikkinen 2016 [80]	Finland	2000–2007	5877 (75.8)	19,455 (74.5)	22–60	case-control	Population	7
Hadjisavvas 2010 [81]	Cyprus	1999–2005	1103 (25.4)	1173 (25.1)	50–59	case-control	Hospital	7
Lumachi 2010 [82]	Italy	No data	238 (14.3)	255 (7.4)	56	case-control	Population	7
Roddam 2007 [83]	United Kingdom	1987–1990	639 (82.3)	640 (79.7)	36–45	case-control	Population	5
Dinger 2006 [84]	Germany	2004–2005	3587 (69.9)	9076 (80.1)	50.6	case-control	Population	7
Magnusson 1999 [85]	Sweden	1993–1995	3008 (35.5)	3248 (33.0)	50–74	cohort	Population	5
Levi 1996 [86]	Switzerland	1990–1995	206 (37.4)	424 (31.6)	27–75	cohort	Population	6
Traina 1996 [87]	Italy	1992–1994	300 (34.3)	300 (46.0)	<46	case-control	Hospitals	5
Viladiu 1996 [88]	Spain	1986–1993	330 (11.5)	346 (18.5)	<75	case-control	Population	6
La Vecchia 1995 [89]	Italy	1991–1994	1991 (17.5)	1899 (14.2)	23–64	case-control	Population	6
Lipworth 1995 [90]	Greece	1989–1991	820 (4.4)	1548 (4.1)	56.4	case-control	Hospital	6
**E. Middle East countries**								
El Sharif 2021 [91]	Palestine	2016–2017	237 (19.8)	237 (10.5)	54.6 ± 10.9	case-control	Population	7
Alipour 2019 [92]	Iran	2004–2008	99 (34.3)	400 (33.2)	40–75	cohort	Population	8
Abedalrahman 2019 [93]	Iraq	2018	147 (17.0)	151 (11.3)	<30–60+	cohort	Hospital	4
Bardaweel 2019 [94]	Jordan	2017	223 (39.0)	223 (21.5)	18–65	case-control	Clinic	7
	Jemen	2011–2015	105 (49.5)	210 (55.2)	No data	cohort	Population	6
Bashamakha 2019 [95]	Saudi Arabia	2014–2016	214 (43.9)	218 (25.2)	57.0 ± 7.3	cohort	Population	5
Alsolami 2019 [96]	Iran	2014–2016	526 (46.8)	562 (40.7)	<40–60+	case-control	Hospitat	7
Dianatinasab 2017 [97]	Palestine	2014–2015	96 (28.1)	197 (28.9)	18–60	case-control	Hospital	7
Kariri 2017 [98]	Saudi Arabia	2001–2013	92 (58.7)	100 (67.0)	30–65	case-control	Clinic	4
Karim 2015 [99]	Saudi Arabia	2013–2014	58 (62.1)	290 (73.8)	30–69	case-control	Hospital	6
Vaisy 2015 [100]	Iran	2013–2014	228 (72.4)	216 (57.4)	47.6	cohort	Clinic	5
Sepandi 2014 [101]	Iran	2001–2012	197 (57.9)	11,653 (55.8)	26–68	cohort	Hospital	4
Tazhibi 2014 [102]	Iran	1999–2010	216 (63.9)	41 (75.6)	20–75	cohort	Hospital	5
Ehsanpour 2013 [103]	Iran	2011	175 (43.4)	350 (25.4)	<41–60+	cohort	Clinic	5
Ghiasvand 2011 [104]	Iran	2005–2008	442 (66.3)	463 (62.9)	41.2 ± 5.7	case-control	Hospital	5
Tehranian 2010 [105]	Iran	no data	312 (38.8)	312 (18.3)	<25–39	cohort	Population	4
Ozmen 2009 [106]	Turkey	2000–2006	1492 (18.4)	2167 (27.8)	18–70	case-control	Hospital	7
Mahouri 2007 [107]	Iran	2000–2002	168 (18.5)	504 (20.0)	27–92	case-control	Population	6
Beji 2007 [108]	Turkey	2002–2003	405 (23.0	1050 (14.7)	28–72	case-control	Hospital	7
Yavari 2005 [109]	Iran	2004	300 (59.0)	303 (49.2)	24–84	case-control	Hospital	6
Kuru 2002 [110]	Turkey	1998–1999	504 (23.6)	610 (16.9)	49.4	case-control	Hospital	5

**Table 2:** Subgroup analysis.

	Geographical Region				
Outcomes	African Countries	American Countries	Asian Countries	European Countries	Middle East Countries
	**Age at menarche** <12 y vs. ≥12 y				
Sudies N [references]	6 [37,38,39,40,41,43]	7 [47,49,51,52,58,60,62]	9 [63,64,66,68,70,71,72,75,78]	5 [79,80,81,84,87]	9 [91,93,95,98,101,102,104,107,109]
RR (95% CI), *p*.	0.84 (0.53; 1.32), 0.452	1.01 (0.93; 1.10), 0.753	1.35 (0.93; 1.95), 0.110	0.98 (0.42; 2.30), 0.970	1.56 (0.99; 2.46), 0.056
Q, *p*. *I*^2^.	42.39, 0.000. 88.21%	7.70, 0.261. 22.06%	72.19, 0.000. 88.92%	1544.61, 0.000, 99.74%	57.27, 0.000. 86.03%
Beeg’s test: tau b, *z*, *p.*	Not available	−0.14, −0.45, 0.625	0.20, 0.49, 0.624	Not available	0.40, 0.98, 0.327
Egger’s test: b0, t, *p.*	−1.76, −0.46, 0.667	−0.25, −0.17, 0.868	1.70, 1,16, 0.282	10.23, 0.48, 0.664	−1.95, −0.52, 0.620
	**Parity** nulliparous/parous				
Studies. N [references]	6 [37,38,39,40,41,43]	7 [47,49,51,52,53,54,60,62]	10 [63,66,68,69,70,71,72,75,77,78]	6 [79,80,81,83,85,86]	10 [91,94,98,101,102,104,106,107,109,110]
RR (95% CI), *p*.	2.21 (1.31; 3.71), 0.003	1.09 (0.85; 1.38), 0.499	1.08 (0.74; 1.57), 0.669	1.34 (1.26; 1.42), 0.000	1.40 (0.93; 2.10), 0.104
Q, *p*. *I*^2^.	41.36, 0.000. 87.91%	41.75, 0.000. 85.63%	98.26, 0.000. 90.84%	5.21, 0.390. 4.13%	68.70, 0.000. 86.90%
Beeg’s test: tau b, *z*, *p.*	Not available	−0.33, −0.52, 0.602	0.00, 0.00, 1.000	−0.07, −0.19, 0.851	0.00, 0.00, 1.000
Egger’s test: b0, t, *p.*	3.42, 0.63, 0.560	3.72, 1.45, 0.208	0.63, 0.33, 0.747	−0.48, −0.53, 0.621	3.14, 2.19, 0.060
	**Breastfeeding** no/yes				
Studies. N [references]	4 [37,38,41,43]	9 [47,49,51,52,53,54,58,60,62]	10 [63,64,68,69,71,72,75,76,77,78]	4 [79,81,84,85]	10 [91,94,95,96,98,99,106,107,109,110]
RR (95% CI), *p*.	2.11 (1.07; 4.15), 0.030	1.12 (1.00; 1.26), 0.047	1.86 (1.40; 2.49), 0.000	1.18 (0.91; 1.53), 0.208	1.88 (1.19; 2.96), 0.007
Q, *p*. *I*^2^.	19.28, 0.000. 84.44%	22.97, 0.003. 65.17%	67.06, 0.000. 86.58%	46.30, 0.000. 93.52%	55.65, 0.000.82.03%
Beeg’s test: tau b, *z*, *p.*	Not available	−0.06, −0.21, 0.835	0.33, 0.94, 0.348	Not available	0.51, 2.06, 0.040
Egger’s test: b0, t, *p.*	−3.22, −0.55, 0.639	1.13, 0.67, 0.524	1.02, 0.63, 0.548	−6.26, −1.25, 0.337	−2.28, −0.87, 0.408
	**Body mass index (BMI)** kg/m^2^				
Studies. N [references]	4 [37,38,39,40]	3 [47,49,60]	3 [66,71,72]	2 [80,81]	4 [96,101,104,110]
			**BMI** 26–29/≤25		
RR (95% CI), *p*.	1.42 (1.10; 1.83), 0.008	1.34 (1.05; 1.47), 0.505	1.71 (0.64; 4.59), 0.288	0.93 (0.74; 1.18), 0.570	1.05 (0.77; 1,41), 0.770
Q, *p*. *I*^2^.	5.41, 0.144. 44.51%	2.84, 0.241. 29.71%	27.53, 0.000. 92.79%	4.98, 0.024. 79.93%	8.60, 0.035. 65.13%
Beeg’s test: tau b, *z*, *p.*	0.33, 0.68, 0.497	−0.33, −0.52, 0.602	Not available	Not available	Not available
Egger’s test: b0, t, *p.*	4.48, 1.73, 0.226	−4.24, −0.95, 0.515	8.86, 1,01, 0.495	Not available	5.43, 2.56, 0.125
			**BMI** ≥30/≤25		
RR (95% CI), *p*.	2.25 (1.07; 4.74), 0.033	1.00 (0.88; 1.14), 0.971	2.18 (0.86; 5.44), 0.103	1.04 (0.73; 1.50), 0.815	1.64 (0.73, 3.68). 0.233
Q, *p*. *I*^2^.	41.29, 0.000. 92.73%	3.16, 0.206. 36.67%	8.90, 0.012. 77.53%	9.45, 0.002. 89.42%	57.01, 0.000. 94.74%
Beeg’s test: tau b, *z*, *p.*	Not available	−0.33, −0.52, 0.602	Not available	Not available	Not available
Egger’s test: b0, t, *p.*	8.44, 1.30, 0.323	−1.57, −0.37, 0.775	2.86, 2.08, 0.286	Not available	17.34, 9.84, 0.018
	**Cigarette smoking** yes/no				
Studies. N [references]	2 [37,38]	3 [54,58,60]	7 [63,66,68,71,76,77,78]	3 [80,81,83]	5 [92,94,96,106,107]
RR (95% CI), *p*.	2.36 (0.65; 8.58), 0.193	1.04 (0.89; 1.27), 0.592	1.37 (0.95; 1.98), 0.089	1.04 (0.91; 1.19), 0.524	1.40 (0.75; 2.62), 0.297
Q, *p*. *I*^2^.	4.24, 0.039. 74.43%	6.07, 0.048. 67.05%	15.36, 0.018. 60.95%	4.50, 0.105. 55.59%	33.79, 0.000. 88.16%
Beeg’s test: tau b, *z*, *p.*	Not available	Not available	−0.48, −0.98, 0.327	Not available	Not available
Egger’s test: b0, t, *p.*	Not available	−4.30, −0.39, 0.763	0.37, 0.29, 0.780	2.08, 2.66, 0.229	2.91, 1.83, 0.164
	**Family history of breast cancer** yes/no				
Studies. N [references]	4 [37,39,40,43]	8 [47,49,50,51,58,60,61,62]	11 [63,64,66,68,69,71,72,74,75,76,78]	5 [80,81,83,84,86]	12 [91,92,93,95,96,98,101,104,106,107,109,110]
RR (95% CI), *p*.	3.90 (2.80; 5.43), 0.000	1.78 (1.51; 2.10), 0.000	1.85 (1.27; 2.69), 0.001	2.01 (1.35; 3.01), 0.001	1.80 (1.21; 2.68), 0.004
Q, *p*. *I*^2^.	2.50, 0.475. 0.00%	29.46, 0.000. 76.24%	57.80, 0.000. 82.72%	94.13, 0.000. 95.75%	80.96, 0.000. 85.18%
Beeg’s test: tau b, *z*, *p.*	−0.33, −0.68, 0.497	−0.14, −0.45, 0.652	0.33, 1,25, 0.211	Not available	0.20, 0.80, 0.421
Egger’s test: b0, t, *p.*	−6.89, −0.77, 0.528	−0.97, −0.46, 0.662	1.92, 2,03, 0.073	2.32, 0.57, 0.606	1.24, 0.67, 0.513

Abbreviations: CI, confidence interval; I^2^, coefficient of inconsistency; N, number of studies; RR, relative risk; *p*, probability value.

## Data Availability

No new data were created or analyzed in this study. Data sharing is not applicable to this article.
